# Possible oral manifestation after vaccination against COVID-19: a case report

**DOI:** 10.1093/omcr/omac136

**Published:** 2022-12-16

**Authors:** Artak Heboyan, Mohmed Isaqali Karobari, Anand Marya

**Affiliations:** Department of Prosthodontics, Faculty of Stomatology, Yerevan State Medical University after Mkhitar Heratsi, Yerevan, Armenia; Department of Conservative & Endodontics, Faculty of Dentistry, University of Puthisastra, Phnom Penh, Cambodia; Center for Transdisciplinary Research, Saveetha Dental College, Saveetha Institute of Medical and Technical Science, Saveetha University, Chennai, India; Department of Orthodontics, Faculty of Dentistry, University of Puthisastra, Phnom Penh, Cambodia; Department of Orthodontics, Faculty of Dental Medicine, Universitas Airlangga, Surabaya, Indonesia

## Abstract

The coronavirus disease 2019 (COVID-19) vaccines are not absolutely safe, and side effects might include oral manifestations, such as rash on the mucous membrane of the mouth and gingival hypertrophy. A 34-year-old male presented to the university dental clinic with malaise, high fever, weakness, tender gums, gingival hypertrophy, rashes on the mucous membrane of the oral cavity and halitosis. Dental professionals must be able to identify and differentiate between lesions of different varieties. This manifestation may be a new feature that can be checked during the history recording and examination part of treatment for patients vaccinated against the severe acute respiratory syndrome coronavirus 2 virus.

## CASE DESCRIPTION

Various vaccines have been developed in a short period to stop the rapid growth of the coronavirus disease 2019 (COVID-19) pandemic [1]. Meanwhile, adverse reactions of the vaccines to the body have not yet been thoroughly studied and elucidated since there are no findings from long-term clinical trials [2].

**Figure 1:**
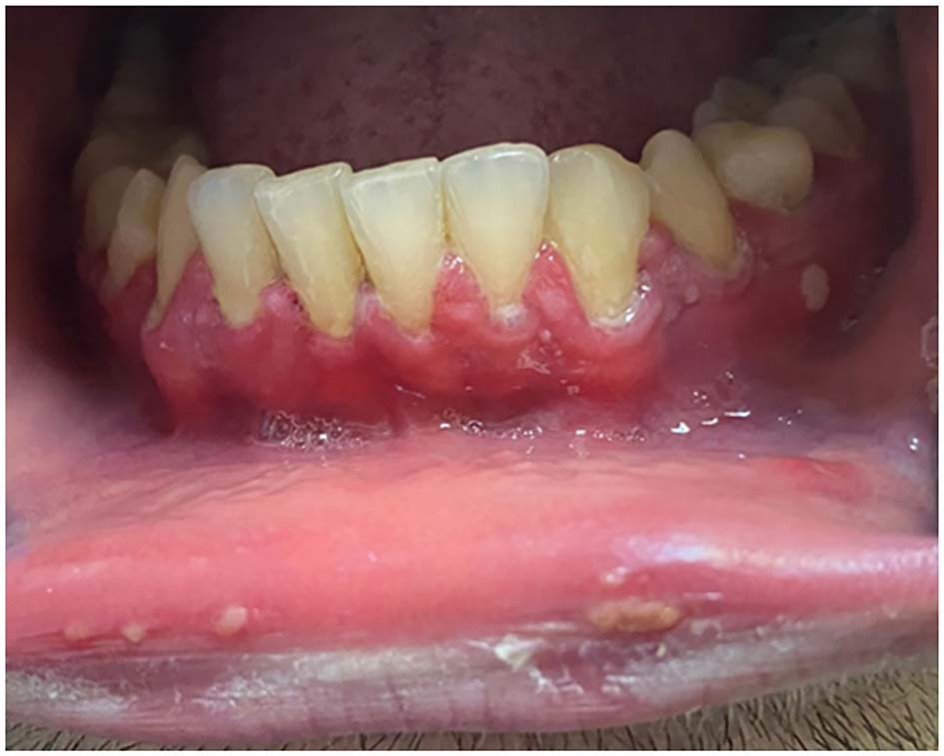
Gingival examination depicts edema and hypertrophic lesions on the boundary between the Vermilion border, the mucous membrane and the gingival region of teeth 34–35.

A 34-year-old male patient presented to the university dental clinic with malaise, high fever, weakness, tender gums, gingival hypertrophy, rashes on the mucous membrane of the oral cavity and halitosis. The man had no previous history of oral conditions and it was noted that the symptoms occurred the day after receiving the second dose of the Moderna COVID vaccine. According to the history recorded, his previous dose was administered 6 months before receiving the second dose. It should be noted that no concomitant somatic pathology was observed in the history. The patient took no medications and was a non-smoker with no long-term adverse habits. Objective examination revealed lesions on the boundary between the Vermilion border, the mucous membrane and the gingival area of teeth 34–35 (Fig. 1). The gingiva in the area of the anterior teeth was edematous. The teeth were covered with dental plaque, as the patient could not brush his teeth due to tender gingiva. A mouth rinse with 0.12% chlorhexidine solution was prescribed to improve oral hygiene, and the patient was asked to use it twice a day at 12-h intervals [3]. Other treatment options could have been antibiotics such as Erythromycin but was not prescribed as there were no other associated symptoms of infection [4]. The possible differential diagnosis for the lesion included drug-induced conditioned enlargement or undiagnosed systemic disorders for which the patient was asked to consult a physician.

While this may not be a severe adverse effect resulting from the vaccination, it can be considered one that must be explored and studied further during long-term clinical trials. Many efforts have been made to study the oral manifestations of COVID-19 infections, and a similar effort is needed to explore the potential oral effects of vaccination [5, 6].

## CONSENT

Written informed consent was obtained from the patient to publish this report following the journal’s patient consent policy.

## CONFLICT OF INTEREST STATEMENT

None declared.
